# Errata for trial publications are not uncommon, are frequently not trivial, and can be challenging to access: a retrospective review

**DOI:** 10.5195/jmla.2019.629

**Published:** 2019-04-01

**Authors:** Kelly Farrah, Danielle Rabb

**Affiliations:** Research Information Specialist, Canadian Agency for Drugs and Technologies in Health (CADTH), Ottawa, ON, Canada, KellyF@cadth.ca; Manager, Research Information Services, Canadian Agency for Drugs and Technologies in Health (CADTH), Ottawa, ON, Canada, DanielleR@cadth.ca

## Abstract

**Objective:**

The research sought to determine the prevalence of errata for drug trial publications that are included in systematic reviews, their potential value to reviews, and their accessibility via standard information retrieval methods.

**Methods:**

The authors conducted a retrospective review of included studies from forty systematic reviews of drugs evaluated by the Canadian Agency for Drugs and Technologies in Health (CADTH) Common Drug Review (CDR) in 2015. For each article that was included in the systematic reviews, we conducted searches for associated errata using the CDR review report, PubMed, and the journal publishers’ websites. The severity of errors described in errata was evaluated using a three-category scale: trivial, minor, or major. The accessibility of errata was determined by examining inclusion in bibliographic databases, costs of obtaining errata, time lag between article and erratum publication, and correction of online articles.

**Results:**

The 40 systematic reviews included 127 articles in total, for which 26 errata were identified. These errata described 38 errors. When classified by severity, 6 errors were major; 20 errors were minor; and 12 errors were trivial. No one database contained all the errata. On average, errata were published 211 days after the original article (range: 15–1,036 days). All were freely available. Over one-third (9/24) of online articles were uncorrected after errata publication.

**Conclusion:**

Errata frequently described non-trivial errors that would either impact the interpretation of data in the article or, in fewer cases, impact the conclusions of the study. As such, it seems useful for reviewers to identify errata associated with included studies. However, publication time lag and inconsistent database indexing impair errata accessibility.

## INTRODUCTION

It is uncertain how often errata for study publications contain information that is valuable for systematic reviews and whether they are retrieved through typical systematic review literature search methods. An erratum is a published note that corrects one or more errors or omissions in an earlier published journal article [1]. Previous studies examining the content of errata have found that the majority of errors reported are minor or trivial mistakes, while a smaller portion describe more serious errors that would affect the interpretation of the study results. Estimates of the proportion of significant errors ranges from 6% [2] to 14% [3] to 24% [4], although these studies differed in how they defined “major” errors as well as in the number and type of journals they included. According to one study, major errors are propagated in other publications, albeit at a lower rate, even after errata have been issued [3].

We identified only 1 previous study that considered errata in the context of systematic reviews [5]. Royle and Waugh concluded that although only 5% of the errata in their study contained information that could change the conclusions of a meta-analysis, most errata would be useful for reviewers in order to facilitate their analyses of the articles’ data [5]. Because their sample consisted of 100 randomized controlled trials already known to have errata, not trials drawn from systematic reviews, the authors were unable to determine the prevalence of errata associated with studies that are included in systematic reviews. A further limitation to that study is that it relied on a single bibliographic database to identify errata.

There is little research on the identification and retrieval of errata. The Cochrane Handbook discusses searching for errata when updating a systematic review in section 6.4.10, “Identifying fraudulent studies, other retracted publications, errata and comments” [6]. It recommends searching for the most recent MEDLINE citation for all included studies in the earlier review to identify errata that may have been subsequently published [6]. MEDLINE and other biomedical databases can either index published errata as separate database records, link the separate erratum record to the original article record, update the original article record to note that an erratum has been issued, or take a combination of these actions. However, indexing of errata may not be complete [4]. For example, in order for an erratum to be included in MEDLINE, “the erratum must appear on a numbered page in an issue of the journal that published the original article” [1]. Further, the National Library of Medicine (NLM) does not assign Medical Subject Heading (MeSH) terms in MEDLINE to errata records [1], which could make them more challenging to identify.

The objectives of this study were: (1) to estimate the frequency of errata for drug trial publications that are included in systematic reviews conducted by the Canadian Agency for Drugs and Technologies in Health (CADTH) Common Drug Review (CDR), (2) to evaluate the errata’s potential impact on reviews by rating the severity of errors they describe, and (3) to determine whether errata are accessible to reviewers through standard systematic review searching methods.

## METHODS

### Systematic reviews and included studies

The authors conducted a retrospective review of the included studies from forty systematic reviews of drugs evaluated by CADTH’s CDR in 2015. A full list of the analyzed reviews is provided in the supplemental appendix. For each systematic review, we extracted all unique journal articles from the included studies list.

### Errata identification

For each unique journal article identified in the reviews’ included study lists, we conducted a search for associated errata using three sources:

1. Errata identified in the CDR reviews: We scanned the publicly accessible CDR clinical review reports to determine any errata that were identified as part of the review process. The reviews involve a systematic literature search. Additionally, as part of the CDR process, manufacturers are required to identify trials and associated publications for the drug under review, as well as any errata related to these publications.
2. PubMed for linked errata: We used variants of author names and publication titles in the PubMed advanced search to identify records for each of the included journal articles. We then scanned the PubMed record for each article to identify information on any associated errata.
3. The journal publisher’s website: We searched for variants of author names and publication titles in the appropriate journal’s website for each article. We scanned these results and the page including the original article to identify information on any associated errata.

For the purposes of this study, we defined an erratum as a published notice correcting an error (or errors) in a previously published journal article [1]. Consistent with NLM, we made no distinction between the terms: errata, corrections, or corrigenda [1]. Accordingly, we included errors regardless of whether they occurred during the publication process or were made by the article authors.

### Errata frequency

We determined the frequency of errata for the published articles that were included in the systematic reviews using the total number of errata identified divided by the total number of journal articles on the included study lists.

### Errors and error severity

We tabulated the total number of unique errors in each erratum. One erratum could report on multiple errors. We evaluated the severity of each error using a three-category scale:

- Major: Error impacts the analysis or interpretation of a primary outcome and, subsequently, the conclusions of the study.
- Minor: Error impacts the analysis or interpretation of an outcome of the study but does not impact the conclusions of the study.
- Trivial: Error does not relate to the analysis or interpretation of any study outcomes or conclusions.

Both authors independently rated each erratum, and conflicts were resolved through discussions with a methodologist at CADTH.

### Errata accessibility

We determined the accessibility of errata by examining:

1. The inclusion of errata citations in MEDLINE, Embase, the Cochrane Central Register of Controlled Trials (CENTRAL), and Scopus: We searched each database using the specific erratum citation information to identify if errata were indexed as separate records. We also searched each database for the original journal article record to determine if it was updated to note that an erratum had been published.
2. The costs of obtaining errata: We determined cost by searching each erratum on the journal publisher’s website to see if the full text of the erratum was freely available or if charges applied for obtaining full text.
3. The time lag between original article publication and erratum publication: We determined the dates using the journal publisher’s website and selected the earliest identified publication date, including any time available as prepublication.
4. Updating of the original publication: We searched the journal publisher’s website to identify if any online versions of the original article had been updated after the publication of the corresponding erratum. If the erratum itself stated that the online version was updated, we did not further verify the online version.

## RESULTS

### Errata frequency

A total of 26 errata were identified for the 127 articles included in the 40 systematic reviews. Of these included articles, 19% (24 out of 127) had an associated erratum (2 articles had 2 separate errata issued).

### Errors and error severity

A total of 38 errors were reported across the 26 errata, giving an average of 1.5 errors per erratum. Of the 38 errors, 6 (15.8%) were classified as major, 20 (52.6%) as minor, and 12 (31.6%) as trivial. Table 1 provides examples of errors in each of these categories.

**Table 1:**
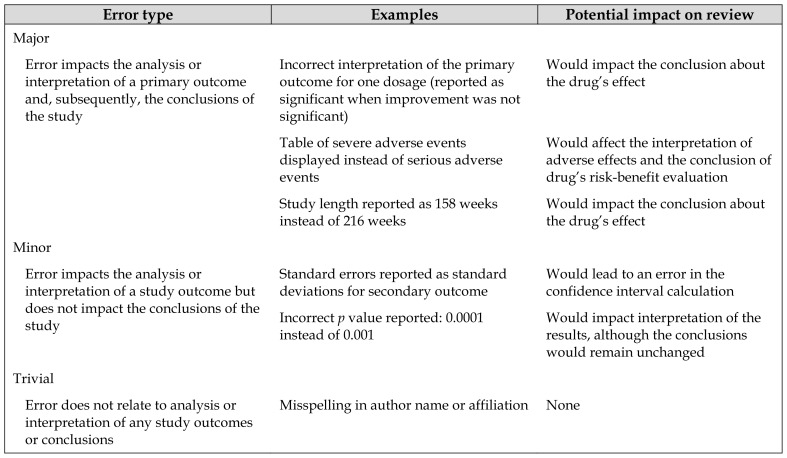
Types of errors found and their impact on interpreting study results

Error type	Examples	Potential impact on review
Major		
Error impacts the analysis or interpretation of a primary outcome and, subsequently, the conclusions of the study	Incorrect interpretation of the primary outcome for one dosage (reported as significant when improvement was not significant)	Would impact the conclusion about the drug’s effect
	Table of severe adverse events displayed instead of serious adverse events	Would affect the interpretation of adverse effects and the conclusion of drug’s risk-benefit evaluation
	Study length reported as 158 weeks instead of 216 weeks	Would impact the conclusion about the drug’s effect
Minor		
Error impacts the analysis or interpretation of a study outcome but does not impact the conclusions of the study	Standard errors reported as standard deviations for secondary outcome	Would lead to an error in the confidence interval calculation
	Incorrect *p* value reported: 0.0001 instead of 0.001	Would impact interpretation of the results, although the conclusions would remain unchanged
Trivial		
Error does not relate to analysis or interpretation of any study outcomes or conclusions	Misspelling in author name or affiliation	None

### Errata accessibility

#### Inclusion in databases

None of the four databases included all twenty-six errata as separate records or updated all original article records to indicate that a corresponding erratum had been published (Table 2). Six errata were not found in any of the four databases and were identified only by searching the publishers’ websites. Although one of these errata was included in several databases as a letter, the records gave no indication that the letter contained a correction. Altogether, the six unindexed errata described seven errors: two major and five minor.

**Table 2:**
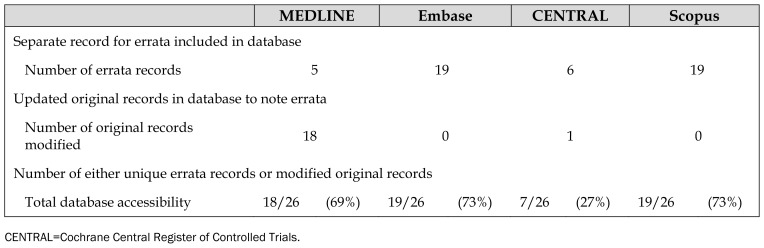
Database accessibility of errata

	MEDLINE	Embase	CENTRAL	Scopus
Separate record for errata included in database				
Number of errata records	5	19	6	19
Updated original records in database to note errata				
Number of original records modified	18	0	1	0
Number of either unique errata records or modified original records				
Total database accessibility	18/26 (69%)	19/26 (73%)	7/26 (27%)	19/26 (73%)

CENTRAL=Cochrane Central Register of Controlled Trials.

Embase and Scopus included the highest number of separate records for errata themselves (19/26). MEDLINE was the only database that frequently updated original records to note that an erratum was issued (18/26). Few of the errata (5/26) had separate records in MEDLINE; however, MEDLINE only began including errata as separate records in 2015 [7].

#### Time lag

On average, errata were published 211 days (median 123 days) after the original article publication. The time lag ranged from 15 days to 1,036 days. The mean time lag for errata correcting a major error was 333 days (median 237 days, n=6) compared to 175 days (median 120 days, n=20) for those including only minor or trivial errors.

#### Cost of obtaining errata

All errata were freely available.

#### Updating of online articles

In total, fourteen of the twenty-four unique journal articles had updated online versions that corrected the errors. Nine uncorrected original articles were found on the publishers’ websites, although all of these did provide a link to the erratum on the article web page. In the remaining case, it was unclear as to whether or not the original version was updated as the erratum did not clearly describe the errors.

## DISCUSSION

### Errata frequency

Just under 1 in 5 articles included in the systematic reviews studied had associated errata. This errata prevalence was higher than estimates in previous studies: 4% in Molckovsky et al.’s review of oncology journals [3], 4% in Hauptman et al.’s review of general medicine and cardiovascular journals [4], and just under 2% in Castillo et al.’s review of imaging journals [2]. The higher percentage in our sample might be due to differences in the types of articles that were assessed. The researchers in the 3 previous studies hand-searched journals and identified errata for all articles found in these journals [2–4]. Our sample was restricted to studies included in systematic reviews of drug trials, which tend to be randomized controlled trials published in high-impact journals and have many authors. Hauptman et al. found a strong positive association between a journal’s impact factor and the rate of errata it issued, as well as a positive correlation between the number of authors an article had and the number of errors found in a variety of article sections [4].

### Severity of errors

The percentage of errors that potentially impacted the conclusions of a study was comparable to the results reported by Molckovsky et al. in 2011 [3] and by Royle and Waugh in 2004 [5]. We found 16% of errors in errata would impact the analysis or interpretation of a study’s primary outcome and, subsequently, the conclusions of the study. Similarly, Molckovsky et al. classified 14% (26/190) of errors identified in high-impact oncology journals as “serious” (as opposed to “trivial”) [3], while Royle and Waugh reported that 15% of errata might affect either (1) the results of a meta-analysis (5%) or (2) the interpretation of the trial only (10%) [5]. Despite the small percentage that changed the results of a meta-analysis, they concluded that the majority of errata would be still be useful for individuals conducting systematic reviews as “full and accurate data can reduce confusion and save reviewers time” [5].

### Errata accessibility

Incomplete indexing and a variable time lag between the initial publication of the article and the notice of erratum can complicate the retrieval of errata for systematic reviews. Not all errata were accessible in the four databases that we searched, with six out of twenty-six only found by searching journal publishers’ websites. Further, when errata were included as separate records in a database, they frequently had minimal information that would facilitate their retrieval through a literature search (i.e., no subject headings, one-word titles, and no abstracts). Of the databases that were studied, only MEDLINE updated an original article’s record to reflect the later publication of an erratum. As a consequence, reviewers might not be aware that an erratum exists when screening records.

There was also a wide range in the amount of lag time between publication of the original article and the erratum publication date—anywhere from 15 to 1,036 days—making it difficult to predict when errata would typically be issued. Further, previous studies have found that time to publication of an erratum was significantly longer for those correcting major errors compared to non-major errors, including Royle and Waugh (mean 7.1 months versus 2.9 months) [5] and Molckovsky et al. (median 8.3 months versus 3.5 months) [3].

One apparent improvement in accessibility since Royle and Waugh’s study [5] was that all the errata in the present study were available in full text free of charge, in contrast to their findings in 2004 that 7 out of 15 journals did not provide free access to errata [5]. However, the updating of online versions of articles remained incomplete. As with Hauptman et al. [4], we found it difficult to identify in some cases whether corrections had been made to the online versions of the journal articles after errata had been issued, although we found a higher number had been corrected (58% in the present study versus 49% in Hauptman et al.).

### Limitations

This research was based on a small sample of errata from single-drug systematic reviews, which could limit the generalizability of the results. It is possible that the frequency and timing of errata vary based on the subject matter and impact factor of journals. We did not evaluate whether the major errors that were identified would have altered the conclusions of the systematic reviews, only whether they would affect conclusions of the original articles. Future research should prospectively evaluate whether errata are retrieved in systematic search results and whether errata records are retrieved in search alerts.

### Implications for practice

Given our findings, evidence from previous studies, and our own experiences as information specialists, we present the following recommendations for searchers, journal publishers, and database producers to improve identification and retrieval of errata.

#### Suggestions for searchers

Those conducting systematic searches are advised to check their bibliographic software settings to confirm that errata information from database records are importing correctly and displaying prominently in citation management software or screening software. For example, in PubMed, the erratum information is contained in a record’s EIN field. Once they have uploaded information into a citation software program, searchers should check how it is displayed for reviewers who are screening titles and abstracts. If this information imports into an area hidden from the reviewers who are screening articles, evidence of the existence of errata can be lost. Further, errata with separate database records may appear to be duplicates of the original article records.

When updating systematic reviews, searchers should follow the guidelines in the Cochrane Handbook [6] for locating errata. Also, searching for errata after the completion of a systematic literature search may be warranted to identify all errata associated with included studies.

#### Suggestions for journal publishers

Despite being freely available, it still takes time and effort to locate the full text of errata through journal publishers’ websites. Frequently, no indication is given in the database record as to the nature of the error. Given that many errors are minor or trivial, reviewers could be discouraged from the effort to seek out errata [4]. As such, we suggest that journal publishers provide descriptive titles for errata, which indicate the nature of the errors, and avoid simple one-word titles such as “erratum” or “correction.” In particular, journal publishers should consider flagging major errors. We concur with Hauptman et al. who suggested that editors could rate severity of errors to increase awareness of serious mistakes [4]. To reduce the circulation of uncorrected versions of the article, we recommend that the online versions of articles be updated when an erratum is issued, in addition to a note indicating that a correction has been made. Furthermore, in the full text of the erratum itself, the original and corrected version of the text should be clearly stated; a simple statement of “changes were made” should be avoided.

#### Suggestions for database producers

Ideally, databases should update the original article record with the erratum information, as well as index a separate record for the erratum itself. Updating the original record would be the more useful of the two, since it keeps the article information and erratum information connected in one place. However, separate records for the errata can also be useful, as they can come up in search alerts, whereas updates to the original article record might not. Furthermore, separate records allow easier citation of errata.

Perhaps the most helpful change that databases could make would be to include the full text or first paragraph of errata in the abstract field. Doing so would save reviewers the time of tracking down the full text and would immediately indicate the nature of the error. This change could be practical given that most errata are fairly short and freely available. Databases should also flag major errors when the errors occur in the abstracts of journal articles and update the abstracts as necessary.

## CONCLUSION

Almost 1 in 5 articles included in the systematic reviews on drugs had an associated erratum. While about a third of errors were considered trivial, more than half of errors were minor ones that could create confusion in interpreting the study data without impacting the conclusions of the study. A smaller portion (16%) were major errors that could affect interpretation of the study’s conclusions. As such, it would be useful to retrieve all associated errata related to articles that are included in a systematic review. Although all errata were freely available, the retrieval of errata is hindered by incomplete inclusion in bibliographic databases, inconsistent updating of the original online version of the article on publishers’ websites, and variable time lags between publication of the original article and of the erratum. Consequently, errata are not fully accessible through standard systematic review searching methods.

## SUPPLEMENTAL FILE

Appendix Systematic reviews included
