# Supplementary material for: Errata for trial publications are not uncommon, are frequently not trivial, and can be challenging to access: a retrospective review
Source: J Med Libr Assoc. 2019 Apr 1;107(2):187–93. doi: 10.5195/jmla.2019.629 (PMC6466495; doi:10.5195/jmla.2019.629)
Supplement: Appendix [file jmla-107-187-s001.pdf]

JMLA Journal of the Medical Library Association

SC

Supplemental content to
J Med Libr Assoc. Apr;107(2):dx.doi.org/10.5195/jmla.2019.629
www.jmla.mlanet.org
© Farrah, Rabb 2019

Errata for trial publications are not uncommon, are frequently not trivial, and can be challenging to access: a retrospective review

Kelly Farrah, MLIS, AHIP; Danielle Rabb, MLIS

APPENDIX

Systematic reviews included

Note: Canadian Agency for Drugs and Technologies in Health (CADTH) Common Drug Review submissions that were evaluated by the Canadian Drug Expert Committee in 2015 were considered for inclusion. While all drug submissions included were evaluated by CADTH's Canadian Drug Expert Committee in 2015, publication of the final review reports ranged from April 2015 to January 2018. Reports on bioequivalent products, reconsiderations of submissions, resubmissions based on a new price, and withdrawn submissions were excluded. Two clinical review reports whose included study lists contained no published journal articles were excluded.

1. Common Drug Review. Clinical review report: ranibizumab (Lucentis) (10 mg/mL solution for intravitreal injection, 0.5 mg dose with monthly re-treatment as needed) for visual impairment due to choroidal neovascularization secondary to pathologic myopia [Internet]. Ottawa, ON, Canada: CADTH; Aug 2015 [cited 18 Jan 2019]. <https://www.cadth.ca/sites/default/files/cdr/clinical/SR0373_LucentisCNV_CL_Report_e.pdf>.
2. Common Drug Review. Clinical review report: rifaximin (Zaxine) (550 mg tablet) for the reduction in risk of overt hepatic encephalopathy (HE) recurrence in patients ≥ 18 years of age [Internet]. Ottawa, ON, Canada: CADTH; Jul 2015 [cited 18 Jan 2019]. <https://cadth.ca/sites/default/files/cdr/clinical/SR0388_Zaxine_CL_Report_e.pdf>.
3. Common Drug Review. Clinical review report: elosulfase alfa (Vimizim) (2 mg/kg of body weight) for long-term enzyme replacement therapy in patients with a confirmed diagnosis of mucopolysaccharidosis IVA (Morquio A syndrome, or MPS IVA) [Internet]. Ottawa, ON, Canada: CADTH; Jul 2015 [cited 18 Jan 2019]. <https://cadth.ca/sites/default/files/cdr/clinical/SR0389_Vimizim_CL_Report_e.pdf>.
4. Common Drug Review. Clinical review report: tocilizumab (Actemra) (162 mg/0.9 mL solution for subcutaneous injection) for adult patients with moderately to severely active rheumatoid arthritis who have inadequate response to one or more DMARDs and/or TNF antagonists [Internet]. Ottawa, ON, Canada: CADTH; Aug 2015 [cited 18 Jan 2019]. <https://cadth.ca/sites/default/files/cdr/clinical/SR0374_ActemraSC_CL_Report.pdf>.
5. Common Drug Review. Clinical review report: pasireotide (Signifor) (0.3 mg/mL, 0.6 mg/mL and 0.9 mg/mL injection) for treatment of adult patients with Cushing disease for whom surgery is not an option or for whom surgery has failed, as long as clinical benefit is derived [Internet]. Ottawa, ON, Canada: CADTH; Aug 2015 [cited 18 Jan 2019]. <https://cadth.ca/sites/default/files/cdr/clinical/SR0372_Signifor_CL_Report.pdf>.
6. Common Drug Review. Clinical review report: everolimus (Afinitor) (oral tablets) for the treatment of patients with subependymal giant cell astrocytoma (SEGA) associated with tuberous sclerosis complex (TSC) that have demonstrated serial growth, who are not candidates for surgical resection and for whom immediate surgical intervention is not required [Internet]. Ottawa, ON, Canada: CADTH; Sep 2017 [cited 18 Jan 2019]. <https://cadth.ca/sites/default/files/cdr/clinical/SR0376_AfinitorSEGA_CL_Report_e.pdf>.

JMLA Journal of the Medical Library Association

SC

Supplemental content to
J Med Libr Assoc. Apr;107(2):dx.doi.org/10.5195/jmla.2019.629
www.jmla.mlanet.org
© Farrah, Rabb 2019

7. Common Drug Review. Clinical review report (resubmission): pirfenidone (Esbriet) (267 mg capsules) for treatment of mild to moderate idiopathic pulmonary fibrosis (IPF) in adults [Internet]. Ottawa, ON, Canada: CADTH; Apr 2015 [cited 18 Jan 2019]. <https://cadth.ca/sites/default/files/cdr/clinical/SR0393_Esbriet_CL_Report_resub.pdf>.

8. Common Drug Review. Clinical review report: eltrombopag olamine (Revolade) 25 mg and 50 mg tablets to increase platelet counts in thrombocytopenic patients with chronic hepatitis C virus (HCV) infection to allow the initiation and maintenance of interferon-based therapy [Internet]. Ottawa, ON, Canada: CADTH; Aug 2015 [cited 18 Jan 2019]. <https://cadth.ca/sites/default/files/cdr/clinical/SR0377_RevoladeTCP_CL_Report.pdf>.

9. Common Drug Review. Clinical review report: ledipasvir/sofosbuvir (Harvoni) for the treatment of chronic hepatitis C virus (CHC) G1 infection in adults [Internet]. Ottawa, ON, Canada: CADTH; Jul 2015 [cited 18 Jan 2019]. <https://cadth.ca/sites/default/files/cdr/clinical/SR0395_Harvoni_CL_Report_e.pdf>.

10. Common Drug Review. Clinical review report: certolizumab pegol (Cimzia) SC for use alone, or in combination with methotrexate (MTX), for reducing signs and symptoms and inhibiting the progression of structural damage as assessed by X-ray in adult patients with moderately to severely active psoriatic arthritis who have failed one or more disease-modifying antirheumatic drugs (DMARDs) [Internet]. Ottawa, ON, Canada: CADTH; Apr 2015 [cited 18 Jan 2019]. <https://cadth.ca/sites/default/files/cdr/clinical/SR0394_CimziaPsA_CL_Report.pdf>.

11. Common Drug Review. Clinical review report: eslicarbazepine acetate (Aptiom) oral tablets for adjunctive therapy in the treatment of partial-onset seizures in patients with epilepsy who are not satisfactorily controlled with conventional therapy [Internet]. Ottawa, ON, Canada: CADTH; May 2016 [cited 18 Jan 2019]. <https://cadth.ca/sites/default/files/cdr/clinical/SR0391_Aptiom_CL_Report.pdf>.

12. Common Drug Review. Clinical review report: lomitapide (Juxtapid) (oral capsules) as an adjunct to a low-fat diet and other lipid-lowering drugs, with or without LDL apheresis, to reduce low-density lipoprotein cholesterol (LDL-C) in adult patients with homozygous familial hypercholesterolemia (HoFH) [Internet]. Ottawa, ON, Canada: CADTH; Jul 2015 [cited 18 Jan 2019]. <https://cadth.ca/sites/default/files/cdr/clinical/SR0386_Juxtapid_CL_Report_e.pdf>.

13. Common Drug Review. Clinical review report: certolizumab pegol (Cimzia) (subcutaneous injection) for reducing signs and symptoms in adult patients with active ankylosing spondylitis (AS) who have had an inadequate response to conventional therapy [Internet]. Ottawa, ON, Canada: CADTH; Sep 2017 [cited 18 Jan 2019]. <https://cadth.ca/sites/default/files/cdr/clinical/SR0385_CimziaAS_CL_Report_e.pdf>.

14. Common Drug Review. Clinical review report: tofacitinib (Xeljanz) tablets for, in combination with methotrexate (MTX), reducing the signs and symptoms of rheumatoid arthritis (RA), in adult patients with moderately to severely active RA who have had an inadequate response to MTX. in cases of intolerance to MTX, physicians may consider the use of Xeljanz (tofacitinib) as monotherapy [Internet]. Ottawa, ON, Canada: CADTH; Jan 2018 [cited 18 Jan 2019]. <https://cadth.ca/sites/default/files/cdr/clinical/SR0380_Xeljanz_CL_Report.pdf>.

15. Common Drug Review. Clinical review report: aflibercept (Eylea) (40 mg/mL solution for intravitreal injection available as a 2 mg single-use vial) for treatment of diabetic macular edema (DME) [Internet]. Ottawa, ON, Canada: CADTH; Jul 2016 [cited 18 Jan 2019]. <https://cadth.ca/sites/default/files/cdr/clinical/SR0396_EyleaDME_CL_Report.pdf>.

Supplemental content to
J Med Libr Assoc. Apr;107(2):dx.doi.org/10.5195/jmla.2019.629
www.jmla.mlanet.org
© Farrah, Rabb 2019

16. Common Drug Review. Clinical review report: apixaban (Eliquis) for treatment of venous thromboembolic events (deep vein thrombosis, pulmonary embolism) and prevention of recurrent deep vein thrombosis and pulmonary embolism [Internet]. Ottawa, ON, Canada: CADTH; Jan 2016 [cited 18 Jan 2019]. <https://cadth.ca/sites/default/files/cdr/clinical/SR0397_EliquisVTE_CL_Report.pdf>.

17. Common Drug Review. Clinical review report: omalizumab (Xolair) (150 mg and 300 mg subcutaneous injection) for treatment of adults and adolescents (12 years of age and above) with chronic idiopathic urticaria who remain symptomatic despite H1 antihistamine treatment [Internet]. Ottawa, ON, Canada: CADTH; Aug 2015 [cited 18 Jan 2019]. <https://cadth.ca/sites/default/files/cdr/clinical/SR0398_XolairCIU_CL_Report.pdf>.

18. Common Drug Review. Clinical review report: apremilast (Otezla) for treatment of adult patients with moderate to severe plaque psoriasis who are candidates for phototherapy or systemic therapy [Internet]. Ottawa, ON, Canada: CADTH; Jul 2015 [cited 18 Jan 2019]. <https://cadth.ca/sites/default/files/cdr/clinical/SR0400_Otezla_CL_Report.pdf>.

19. Common Drug Review. Clinical review report: aflibercept (Eylea) (40 mg/mL solution for intravitreal injection available as a 2 mg single-use vial) for treatment of visual impairment due to macular edema secondary to central retinal vein occlusion (CRVO) [Internet]. Ottawa, ON, Canada: CADTH; Jun 2016 [cited 18 Jan 2019]. <https://cadth.ca/sites/default/files/cdr/clinical/SR0401_EyleaCRVO_CL_Report.pdf>.

20. Common Drug Review. Clinical review report: alemtuzumab (Lemtrada, intravenous) for the management of adult patients with relapsing-remitting multiple sclerosis (RRMS), with active disease defined by clinical and imaging features, who have had an inadequate response to interferon beta or other disease-modifying therapies [Internet]. Ottawa, ON, Canada: CADTH; Jun 2015 [cited 18 Jan 2019]. <https://cadth.ca/sites/default/files/cdr/clinical/SR0405_Lemtrada_RRMS_CL_Report.pdf>.

21. Common Drug Review. Clinical review report: ombitasvir, paritaprevir, ritonavir and dasabuvir (Holkira Pak) for the treatment of adults with genotype 1 chronic hepatitis C virus (HCV) infection, including those with compensated cirrhosis [Internet]. Ottawa, ON, Canada: CADTH; Jun 2015 [cited 18 Jan 2019]. <https://www.cadth.ca/sites/default/files/cdr/clinical/SR0406_HolkiraPak_CL_Report.pdf>.

22. Common Drug Review. Clinical review report: secukinumab (Cosentyx) subcutaneous injection for the treatment of moderate to severe plaque psoriasis in adult patients who are candidates for systemic therapy or phototherapy [Internet]. Ottawa, ON, Canada: CADTH; Oct 2015 [cited 18 Jan 2019]. <https://cadth.ca/sites/default/files/cdr/clinical/SR0407_Cosentyx_CL_Report.pdf>.

23. Common Drug Review. Clinical review report: azelastine hydrochloride and fluticasone propionate nasal spray (AZE/FP) for the symptomatic treatment of moderate-to-severe seasonal allergic rhinitis and associated ocular symptoms in adults and adolescents aged 12 and older for whom monotherapy with either antihistamines or intranasal corticosteroids is not considered sufficient [Internet]. Ottawa, ON, Canada: CADTH; May 2016 [cited 18 Jan 2019]. <https://cadth.ca/sites/default/files/cdr/clinical/SR0408_Dymista_CL_Report.pdf>.

© Farrah, Rabb 2019

24. Common Drug Review. Clinical review report: tiotropium bromide monohydrate (Spiriva Respimat) for oral inhalation for the long-term once-daily maintenance bronchodilator treatment of airflow obstruction in patients with chronic obstructive pulmonary disease (COPD), including chronic bronchitis and/or emphysema, and for the reduction of exacerbations [Internet]. Ottawa, ON, Canada: CADTH; Jul 2015 [cited 18 Jan 2019]. <https://cadth.ca/sites/default/files/cdr/clinical/SR0412_SpirivaRespimat_CL_Report.pdf>.

25. Common Drug Review. Clinical review report: linaclotide (Constella) for treatment of irritable bowel syndrome with constipation (IBS-C) in adults [Internet]. Ottawa, ON, Canada: CADTH; Sep 2015 [cited 18 Jan 2019]. <https://cadth.ca/sites/default/files/cdr/clinical/SR0409_Constella_CL_Report.pdf>.

26. Common Drug Review. Clinical review report: vedolizumab (Entyvio) for treatment of adult patients with moderately to severely active ulcerative colitis who have had an inadequate response, loss of response to, or were intolerant to either conventional therapy or infliximab, a TNF alpha antagonist [Internet]. Ottawa, ON, Canada: CADTH; Oct 2015 [cited 18 Jan 2019]. <https://cadth.ca/sites/default/files/cdr/clinical/SR0421_Entyvio_CL_Report.pdf>.

27. Common Drug Review. Clinical review report: taliglucerase alfa (Elelyso) (30 to 60 U/kg intravenous infusion) for long-term enzyme replacement therapy for adults and children (2 to 17 years old) with a confirmed diagnosis of type 1 Gaucher disease and for hematological manifestations in pediatric patients with a confirmed diagnosis of type 3 Gaucher disease [Internet]. Ottawa, ON, Canada: CADTH; Jan 2018 [cited 18 Jan 2019]. <https://cadth.ca/sites/default/files/cdr/clinical/SR0390_Elelyso_CL_Report.pdf>.

28. Common Drug Review. Clinical review report: umeclidinium bromide (Incruse Ellipta) for long-term, once daily maintenance bronchodilator treatment of airflow obstruction in patients with chronic obstructive pulmonary disease (COPD), including chronic bronchitis and emphysema [Internet]. Ottawa, ON, Canada: CADTH; Jan 2018 [cited 18 Jan 2019]. <https://cadth.ca/sites/default/files/cdr/clinical/SR0422_Incruse_Ellipta_CL_Report.pdf>.

29. Common Drug Review. Clinical review report: daclatasvir (Daklinza) in combination with other agents for the treatment of chronic hepatitis C (CHC) infection in adults with hepatitis C virus (HCV) genotype 1, and 2 infection and compensated liver disease (including cirrhosis) [Internet]. Ottawa, ON, Canada: CADTH; Sep 2015 [cited 18 Jan 2019]. <https://cadth.ca/sites/default/files/cdr/clinical/SR0417_Daklinza_CL_Report.pdf>.

30. Common Drug Review. Clinical review report: nintedanib (Ofev) for the treatment of idiopathic pulmonary fibrosis (IPF) [Internet]. Ottawa, ON, Canada: CADTH; Oct 2015 [cited 18 Jan 2019]. <https://cadth.ca/sites/default/files/cdr/clinical/SR0426_Ofev_CL_Report.pdf>.

31. Common Drug Review. Clinical review report: empagliflozin (Jardiance) for adults with type 2 diabetes mellitus to improve glycemic control in combination with metformin and a sulfonylurea when diet, exercise, and dual therapy (with metformin plus a sulfonylurea) do not provide adequate glycemic control [Internet]. Ottawa, ON, Canada: CADTH; Oct 2015 [cited 18 Jan 2019]. <https://cadth.ca/sites/default/files/cdr/clinical/SR0427_Jardiance_CL_Report.pdf>.

JMLA Journal of the Medical Library Association

SC

Supplemental content to
J Med Libr Assoc. Apr;107(2):dx.doi.org/10.5195/jmla.2019.629
www.jmla.mlanet.org
© Farrah, Rabb 2019

32. Common Drug Review. Clinical review report: dapagliflozin (Forxiga) for use in patients with type 2 diabetes mellitus to improve glycemic control in combination with: Metformin, when the existing therapy, along with diet and exercise, does not provide adequate glycemic control; a sulfonylurea, when the existing therapy, along with diet and exercise, does not provide adequate glycemic control; insulin (alone or with metformin), when the existing therapy, along with diet and exercise, does not provide adequate glycemic control [Internet]. Ottawa, ON, Canada: CADTH; Nov 2015 [cited 18 Jan 2019]. <https://cadth.ca/sites/default/files/cdr/clinical/SR0428_Forxiga_CL_Report.pdf>.

33. Common Drug Review. Clinical review report: ivermectin (Rosiver) for the treatment of inflammatory lesions (i.e., papules and pustules) of rosacea in adults 18 years of age or older [Internet]. Ottawa, ON, Canada: CADTH; Feb 2016 [cited 18 Jan 2019]. <https://cadth.ca/sites/default/files/cdr/clinical/SR0429_Rosiver_CL_Report.pdf>.

34. Common Drug Review. Clinical review report: tolvaptan (Jinarc) to slow the progression of kidney enlargement in patients with autosomal dominant polycystic kidney disease (ADPKD) [Internet]. Ottawa, ON, Canada: CADTH; Nov 2015 [cited 18 Jan 2019]. <https://cadth.ca/sites/default/files/cdr/clinical/SR0435_Jinarc_CL_Report.pdf>.

35. Common Drug Review. Clinical review report: tiotropium bromide monohydrate and olodaterol hydrochloride (Inspiolto Respimat) for oral inhalation for the long-term, once daily maintenance bronchodilator treatment of airflow obstruction in patients with chronic obstructive pulmonary disease (COPD), including chronic bronchitis and emphysema [Internet]. Ottawa, ON, Canada: CADTH; Dec 2015 [cited 18 Jan 2019]. <https://cadth.ca/sites/default/files/cdr/clinical/SR0436_Inspiolto_Respimat_CL_Report.pdf>.

36. Common Drug Review. Clinical review report: riociguat (Adempas) for the treatment of pulmonary arterial hypertension (PAH, World Health Organization [WHO] Group 1), as monotherapy or in combination with endothelin receptor antagonists in adult patients (≥ 18 years of age) with functional class II or III pulmonary hypertension [Internet]. Ottawa, ON, Canada: CADTH; Dec 2015 [cited 18 Jan 2019]. <https://cadth.ca/sites/default/files/cdr/clinical/SR0438_Adempas_CL_Report.pdf>.

37. Common Drug Review. Clinical review report: inhaled fluticasone furoate (Arnuity Ellipta) (100 mcg and 200 mcg) for the treatment of once daily maintenance treatment of steroid responsive bronchial asthma in patients aged 12 years and older [Internet]. Ottawa, ON, Canada: CADTH; Dec 2015 [cited 18 Jan 2019]. <https://cadth.ca/sites/default/files/cdr/clinical/SR0439_Arnuity_Ellipta_CL_Report.pdf>.

38. Common Drug Review. Clinical review report: peginterferon beta-1a (Plegridy — subcutaneous injection) for the treatment of relapsing-remitting multiple sclerosis to reduce the frequency of clinical exacerbations and to slow the progression of disability [Internet]. Ottawa, ON, Canada: CADTH; Dec 2015 [cited 18 Jan 2019]. <https://cadth.ca/sites/default/files/cdr/clinical/SR0440_Plegridy_CL_Report.pdf>.

39. Common Drug Review. Clinical review report: ivacaftor (Kalydeco) for the treatment of cystic fibrosis in patients 18 years of age and older with a R117H mutation of the cystic fibrosis transmembrane conductance regulator (CFTR) gene [Internet]. Ottawa, ON, Canada: CADTH; Jun 2016 [cited 18 Jan 2019]. <https://cadth.ca/sites/default/files/cdr/clinical/SR0430_KalydecoR117H_CL_Report.pdf>.

Journal of the Medical Library Association

Supplemental content to
J Med Libr Assoc. Apr;107(2):dx.doi.org/10.5195/jmla.2019.629
www.jmla.mlanet.org
© Farrah, Rabb 2019

40. Common Drug Review. Clinical review report: rotigotine (Neupro) for the treatment of the signs and symptoms of idiopathic Parkinson's disease. Neupro may be used both as early therapy, without concomitant levodopa, and as an adjunct to levodopa [Internet]. Ottawa, ON, Canada: CADTH; Nov 2015 [cited 18 Jan 2019]. <https://cadth.ca/sites/default/files/cdr/clinical/SR0432_Neupro_CL_Report_resub.pdf>.
